# Neptunium Pyridine Dipyrrolide Complexes

**DOI:** 10.1021/acs.organomet.4c00472

**Published:** 2025-01-09

**Authors:** Leyla R. Valerio, Andrew W. Mitchell, Lauren M. Lopez, Matthias Zeller, Suzanne C. Bart, Ellen M. Matson

**Affiliations:** † Department of Chemistry, University of Rochester, Rochester, New York 14627, United States; ‡ H. C. Brown Laboratory, James Tarpo Jr. and Margaret Tarpo Department of Chemistry, Purdue University, West Lafayette, Indiana 47907, United States

## Abstract

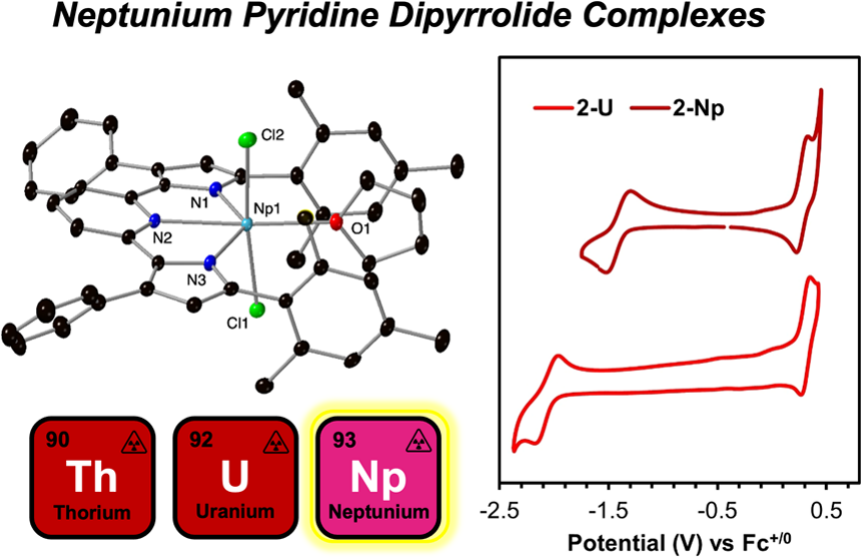

Two pyridine dipyrrolide neptunium(IV) complexes, (^Mes^PDP^Ph^)NpCl_2_(THF) and Np(^Mes^PDP^Ph^)_2_, where (^Mes^PDP^Ph^)^2–^ is the doubly deprotonated form of 2,6-bis(5-(2,4,6-trimethylphenyl)-3-phenyl-1*H*-pyrrol-2-yl)pyridine, have been prepared. Characterization of the complexes has been performed through a combination of solid- and solution-state methods, including single-crystal X-ray diffraction and electronic absorption and nuclear magnetic resonance spectroscopies. Collectively, these data confirm the formation of the mono- and bis-ligated species. Electrochemistry of a series of bis-ligated actinide complexes, An(^Mes^PDP^Ph^)_2_ (An = Th, U, Np), is presented.

## Introduction

The nonaqueous chemistry of actinide ions is motivated by the ability to elucidate fundamental bonding and electronic structure trends not possible in aqueous environments.^1−5^ An advantage of this relatively nonpolar environment is that it facilitates the study of a variety of organic ligands to encapsulate actinide ions in a wide range of oxidation states. Understanding the chemistry of actinides in nonaqueous environments is important in separations chemistry for nuclear fuel reprocessing, where there are commonly aqueous/organic interfaces from which ions must be extracted.^1,2^ The compositional complexity of spent nuclear fuel mixtures warrants a comparison between the electronic properties of isostructural actinide complexes.^6−8^

The majority of nonaqueous actinide studies have focused on U and Th, whereas far less progress has been made for transuranium elements due to their reduced availability and comparatively high specific activities.^1,6^ The chemistry of neptunium (^237^Np) is a burgeoning area in recent years because of its intriguing solution-phase redox chemistry that can span oxidation states of +2 to +7.^9^ Neptunium also lends itself to stabilization by a variety of ligand frameworks that can be modified to tune the electronic properties of the central ion. Early examples include “classic” organometallic ligands such as the cyclopentadienyl (Cp = (C_5_H_5_)^−^) anion and cyclooctatetraene dianion COT (C_8_H_8_^2–^).^9,10^ More recently, such studies have been expanded to include the dipyrrolide, dianionic macrocycle *trans*-calix[2]benzene[2]pyrrolide^2–^, which has been used for the complexation of Th(IV), U(IV), and Np(IV) cations, where the steric protection imparted by the bulky macrocycle has allowed for facile reduction of the uranium and neptunium ions.^11,12^ Sessler and co-workers have additionally described the synthesis and structural characterization of a series of Th(IV), U(IV), and Np(IV) complexes featuring coordination to dipyriamethyrin.^13^ Such series have also been developed in collaboration with Albrecht-Schönzart, where the synthesis of the dioxophenoxazine ligand, DOPO (DOPO = 2,4,6,8-tetra-*tert*-butyl-1-oxo-1*H*-phenoxazine-9-olate), was reported, including derivatives of Th(IV), U(IV), Np(IV), and Pu(IV), which featured ligand radicals due to the highly reducing actinides and redox-active nature of the dioxophenoxazine ligand.^14^

Of late, a subset of tridentate pincer ligands, pyridine dipyrrolides (PDPs), in the dianionic, doubly deprotonated form, have attracted attention as redox-active ligands for transition metal, main group, and actinide elements.^15−28^ Indeed, the reported complexes have been demonstrated to show great promise in the fields of photochemistry and catalysis. For example, the group(IV)-derived photosensitizer Zr(^Mes^PDP^Ph^)_2_, where (^Mes^PDP^Ph^)^2–^ is the doubly deprotonated form of 2,6-bis(5-(2,4,6-trimethylphenyl)-3-phenyl-1*H*-pyrrol-2-yl)pyridine, was reported to have a long-lived triplet excited state that exhibited photoluminescence (Φ_PL_ = 0.45) that could be utilized for photoredox catalysis.^25^ Recent efforts have focused on the isolation of heavier element congeners of the Zr(^Mes^PDP^Ph^)_2_ complex, notably Hf(IV),^29^ Sn(IV),^16^ and Th(IV)^28^ to investigate the effect of heavy atoms on the photophysical properties of the complexes.

Recently, some of us have reported the synthesis and characterization of high-valent uranyl and U(IV) and Th(IV) adducts of the pyridine dipyrrolide ligand class.^27,28^ It was determined that the redox-active PDP ligand is capable of stabilizing various actinide ion oxidation states. An early report details the synthesis of uranyl adducts of the PDP ligand (^Mes^PDP^Ph^)^2–^ and (^Cl2Ph^PDP^Ph^)^2–^.^27,28^ Upon investigation of the electrochemical properties of (PDP)UO_2_(THF) complexes using cyclic voltammetry (CV), reversible U^VI^/U^V^ reduction couples at modest reduction potentials (−1.22 to −1.15 V vs Fc^+/0^) were revealed, suggesting that reduction of the metal ion should be facile. Moreover, the PDP ligand was observed to coordinate midvalent U(IV) and Th(IV) cations, forming complexes with unique optical properties.^28^ The absorption spectra of the uranium derivatives were dominated by ligand-to-metal charge transfer (LMCT) transitions, whereas the thorium complexes exhibited exclusively intraligand charge transfer (ILCT) and ligand-to-ligand charge transfer (LLCT) transitions. The thorium derivatives were also photoluminescent, with long-lived excited states (0.250–0.300 ms) and high quantum efficiencies between 40 and 45%.

Herein, we extend our studies with the PDP ligand from early actinides to neptunium by the synthesis and spectroscopic characterization of (^Mes^PDP^Ph^)NpCl_2_(THF) (**1-Np**) and Np(^Mes^PDP^Ph^)_2_ (**2-Np**). Electrochemical studies of **2-Np** in comparison to the Th and U congeners reveal systematic shifts in the An(IV)/An(III) couples, where Np(III) derivatives of these molecules are more easily accessed due to the less negative overall reduction potential of Np ions (vs Th, U).^30^ From these studies, we aim to develop a series of PDP-actinide compounds that allow us to probe the influence of increasing the f-electron count on their spectroscopic and electrochemical properties.

## Experimental Section

### General Considerations

All air- and moisture-sensitive manipulations with Np were performed using standard Schlenk techniques or in an MBraun negative-pressure argon atmosphere drybox. The MBraun drybox was equipped with a cold well and a −35 °C freezer for cooling samples and crystallizations. All air- and moisture-sensitive manipulations with thorium or uranium were carried out using a standard high-vacuum line, Schlenk techniques, or an MBraun inert atmosphere drybox containing an atmosphere of purified dinitrogen. Solvents for sensitive manipulations were dried and deoxygenated using literature procedures with a Seca solvent purification system or a glass contour solvent purification system (Pure Process Technology, LLC) and stored over activated 4 Å molecular sieves (Fisher Scientific) prior to use. Benzene-*d*_6_ was purchased from Cambridge Isotope Laboratories, degassed by three freeze–pump–thaw cycles, and dried with molecular sieves. NpCl_4_(DME)_2_,^31^ UCl_4_,^32^ ThCl_4_(DME)_2_,^33^ and H_2_(^Mes^PDP^Ph^)^19^ were prepared according to literature procedures. All other chemicals were purchased from commercial sources and used without further purification.

### Safety Considerations

**Caution!**^237^Np represents a health risk due to its α and γ emission and its decay to the short-lived ^233^Pa isotope (*t*_1/2_ = 27.0 days), which is a strong β and γ emitter. All studies with Np were conducted in a laboratory equipped for radioactive materials. All studies were modeled on depleted uranium prior to working with ^237^Np. Depleted uranium (primary isotope ^238^U) is a weak α-emitter (4.197 MeV) with a half-life of 4.47 × 10^9^ years, and ^232^Th is a weak α-emitter (4.082 MeV) with a half-life of 1.41 × 10^10^ years; manipulations and reactions should be carried out in monitored fume hoods or in an inert atmosphere drybox in a radiation laboratory equipped with α and β counting equipment. Due to the radiological hazards associated with working with ^237^Np and the subsequent need to recover neptunium material for recycling measures, elemental analyses of the neptunium complexes reported in this study were not obtained.

### Synthesis of (^Mes^PDP^Ph^)NpCl_2_(THF) (1-Np)

In the glovebox, H_2_^Mes^PDP^Ph^ (0.010 g, 0.017 mmol, 1 equiv) was dissolved in approximately 2 mL of toluene in a 20 mL scintillation vial equipped with a magnetic stirrer. In a separate vial, LiHMDS (0.006 g, 0.034 mmol, 2.05 equiv) was dissolved in 2 mL of toluene and added dropwise to the stirring H_2_^Mes^PDP^Ph^ solution, to make Li_2_^Mes^PDP^Ph^. The resultant solution was stirred for 1 h. In a third 20 mL scintillation vial, NpCl_4_(DME)_2_ (0.010 g, 0.018 mmol, 1 equiv) was dissolved in ∼0.5 mL of THF, and to it was added the Li_2_^Mes^PDP^Ph^ solution while stirring. The reaction was stirred at room temperature for 2 h, and the resultant red solution was filtered over Celite with a glass microfiber plug and dried *in vacuo*. The solid was triturated with pentane until washings ran clear and dried again, producing the title compound. Yield: 63%. Red single crystals were obtained by vapor diffusion of pentane into a saturated solution of the product in toluene at −30 °C. ^1^H NMR (500 MHz, C_6_D_6_) δ 4.48 (4*H*), 4.22 (4*H*), 0.09 (5*H*), −0.23 (5*H*), −0.55 (5*H*), −0.90 (8*H*), −2.04 (2*H*), −4.33 (12*H*), −9.25 (4*H*), −16.10 (6*H*), −25.00 (4*H*).

### Synthesis of Np(^Mes^PDP^Ph^)_2_ (2-Np)

In the glovebox, H_2_^Mes^PDP^Ph^ (0.021 g, 0.036 mmol, 1 equiv) was dissolved in approximately 2 mL of toluene in a 20 mL scintillation vial equipped with a magnetic stirrer. In a separate vial, LiHMDS (0.012 g, 0.072 mmol, 2.05 equiv) was dissolved in 1 mL of toluene and added dropwise to the stirring H_2_^Mes^PDP^Ph^ solution, to make Li_2_^Mes^PDP^Ph^. The resultant solution was stirred for 1 h. In a third 20 mL scintillation vial, NpCl_4_(DME)_2_ (0.010 g, 0.018 mmol, 0.5 equiv) was suspended in ∼1 mL of toluene and to it was added the Li_2_^Mes^PDP^Ph^ solution while stirring. The reaction was heated at ∼90 °C for 6 h. The product was cooled to room temperature and filtered over a bed of Celite with a glass microfiber plug. The resultant red solution was dried *in vacuo*, triturated with pentane until washings ran clear, and dried again, producing the title compound. Yield: 70%. ^1^H NMR (400 MHz, C_6_D_6_) δ 14.63 (t, *J* = 7.8 Hz, 2H), 13.94 (d, *J* = 8.1 Hz, 4H), 11.06 (s, 4H), 8.34 (d, *J* = 7.5 Hz, 8H), 7.97 (t, *J* = 7.5 Hz, 8H), 7.61 (t, *J* = 7.5 Hz, 4H), 7.06 (s, 8H), 3.28 (s, 12H), −3.40 (s, 24H).

### Synthesis of Np(^Ph^PDP^Ph^)_2_ (3-Np)

In the glovebox, H_2_^Ph^PDP^Ph^ (0.021 g, 0.036 mmol, 1 equiv) was dissolved in approximately 2 mL of toluene in a 20 mL scintillation vial equipped with a magnetic stirrer. In a separate vial, LiHMDS (0.012 g, 0.072 mmol, 2.05 equiv) was dissolved in 1 mL of toluene and added dropwise to the stirring H_2_^Ph^PDP^Ph^ solution, to make Li_2_^Ph^PDP^Ph^. The resultant solution was stirred for 1 h. In a third 20 mL scintillation vial, NpCl_4_(DME)_2_ (0.010 g, 0.018 mmol, 0.5 equiv) was suspended in ∼1 mL of toluene and to it was added the Li_2_^Ph^PDP^Ph^ solution while stirring. The reaction was stirred for 12 h at room temperature. The product was filtered over a bed of Celite with a glass microfiber plug. The resultant red solution was dried *in vacuo*, triturated with pentane until washings ran clear, and dried again, producing the title compound. Yield: 74%. ^1^H NMR (400 MHz, C_6_D_6_) δ 16.09 (d, *J* = 8.1 Hz, 4*H*), 15.43 (t, *J* = 7.8 Hz, 2*H*), 11.34 (s, 4*H*), 9.73 (d, *J* = 7.5 Hz, 8*H*), 8.48 (t, *J* = 7.5 Hz, 8*H*), 8.05 (dt, *J* = 19.3, 7.2 Hz, 8*H*), 7.02 (t, *J* = 6.8 Hz, 4*H*), 6.84 (d, *J* = 6.8 Hz, 8*H*), −14.52 (s, 8*H*).

#### Physical Measurements

^1^H NMR spectra for neptunium compounds were recorded at room temperature on a Bruker AV-III-HD-400 spectrometer operating at 400.13 MHz. All chemical shifts are reported with respect to residual solvent relative to the chosen deuterated solvent as a standard. ^1^H spectra were collected with 0.5 s acquisition time, 0 s delay time, and a sweep width of 200 ppm and for 128 scans. ^1^H NMR spectra for all other compounds (Th, U) were recorded at room temperature on a 400 MHz Bruker AVANCE spectrometer or a 500 MHz Bruker AVANCE spectrometer locked on the signal of deuterated solvents. All chemical shifts are reported relative to the chosen deuterated solvent as a standard. Cyclic voltammetry (CV) on neptunium complexes was performed using a three-electrode setup inside a negative-pressure argon glovebox (MBraun UniLab) using a CH Instruments 620E potentiostat. Cyclic voltammetry (CV) on thorium and uranium complexes was performed using a three-electrode setup inside a nitrogen-filled glovebox (MBraun UniLab) using a Bio-Logic SP 150 potentiostat/galvanostat and the EC-Lab software suite. The concentrations of the complexes and the supporting electrolyte (TBAPF_6_) were kept at 1 and 100 mM, respectively, throughout all measurements. CVs were recorded using a 3 mm diameter glassy carbon working electrode (CH Instruments), a Pt wire auxiliary electrode (CH Instruments), and a silver wire reference electrode with ferrocene used as an internal standard after completion of the measurements. Potentials were then referenced versus the Fc^+/0^ redox couple. Electronic absorption measurements for neptunium were recorded at room temperature in anhydrous dichloromethane or toluene in sealed 1 cm quartz cuvettes using a JASCO V-770 UV–vis–NIR spectrophotometer equipped with a fiber optic stage and sample holder. Electronic absorption measurements for thorium and uranium were recorded at room temperature in anhydrous dichloromethane solution in sealed 1 cm quartz cuvettes using an Agilent Cary 6000i UV–vis/NIR spectrophotometer.

#### X-ray Crystallography

Single crystals suitable for X-ray diffraction were coated with poly(isobutylene) oil in the glovebox and quickly transferred to the goniometer head of a Bruker Quest diffractometer with a fixed chi angle, a sealed tube fine focus X-ray tube, a single-crystal curved graphite incident beam monochromator, a Photon II area detector, and an Oxford Cryosystems low-temperature device. Examination and data collection were performed with Mo Kα radiation (λ = 0.71073 Å) at 150 K.

## Results and Discussion

Targeting a scaled-down synthesis of the monoligand U complex, (^Mes^PDP^Ph^)UCl_2_(THF) (**1-U**), two equivalents of LiN(SiMe_3_)_2_ were added to a solution of H_2_(^Mes^PDP^Ph^) in toluene, affording Li_2_(^Mes^PDP^Ph^) *in situ*. Addition of this solution to one equivalent of UCl_4_ dissolved in a minimal amount of THF resulted in a gradual color change to dark red after stirring at room temperature for about 2 h. Workup of the product and analysis by ^1^H NMR spectroscopy confirmed the formation of **1-U**.

**Figure 1:**
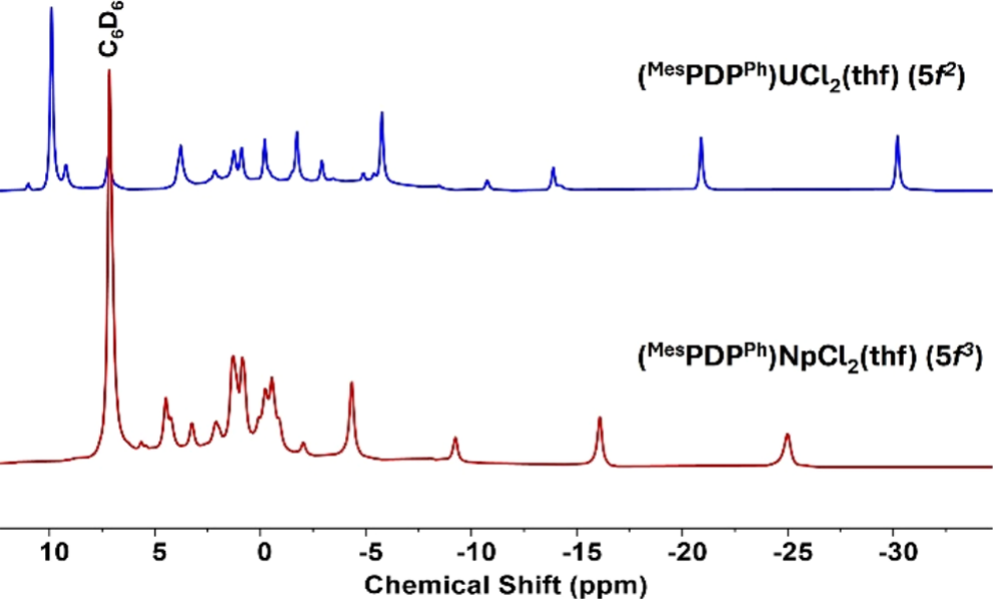
^1^H NMR spectrum (400 MHz) of **1-Np** stacked with **1-U** for comparison collected in C_6_D_6_ at room temperature (∼21 °C). For full assignments, see the Supporting Information.

**Scheme 1:**
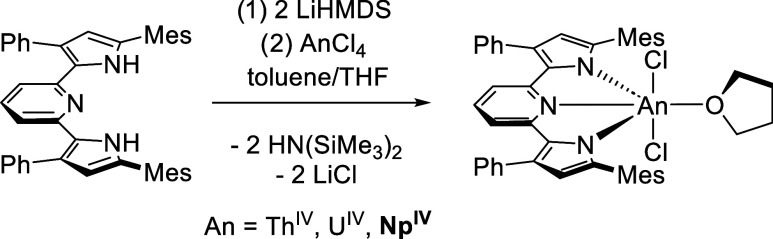
Synthesis of (^Mes^PDP^Ph^)AnCl_2_(THF) Complexes (**1-Th**, **1-U**, **1-Np**)

Following the successful formation of **1-U**, the synthesis of the monoligand PDP Np complex was pursued. The addition of one equivalent of Li_2_(^Mes^PDP^Ph^) in toluene to a solution of NpCl_4_(DME)_2_ in THF results in a color change to dark red after stirring for 2 h at room temperature. Workup of the product afforded (^Mes^PDP^Ph^)NpCl_2_(THF) (**1-Np**) in 63% yield (Scheme 1; see the Experimental Section for additional details). The formation of the monoligated neptunium species was probed by ^1^H NMR spectroscopy. We note that ^13^C NMR spectroscopy was not pursued due to the paramagnetism of the Np ion. The ^1^H NMR spectrum revealed paramagnetically shifted and broadened resonances ranging from +5 to −25 ppm (Figure 1); the overall pattern of resonances is similar to that reported for **1-U**. Analysis of the integrations in the ^1^H NMR spectrum revealed resonances at −4.33 and −16.10 ppm with relative integrations of 12H to 6H. These signals are assigned to the *ortho*-methyl and *para*-methyl groups of the mesityl substituent of the PDP ligand. Interestingly, these resonances are shifted significantly upfield in comparison to those reported for **1-U** (9.75 and −5.59 ppm, respectively), indicating that the protons are shielded to a greater degree with the Np(IV), *f*^3^ ion coordinated. The resonance corresponding to the pyrrole protons, typically used as a spectroscopic handle for PDP compounds, was located at −2.04 ppm with a relative integration of 2H. The remaining resonances in the ^1^H NMR spectrum have relative integrations of 4H, but they are not baseline resolved or are overlapping with one another, making integration ineffective and precluding definitive assignments.

**Figure 2:**
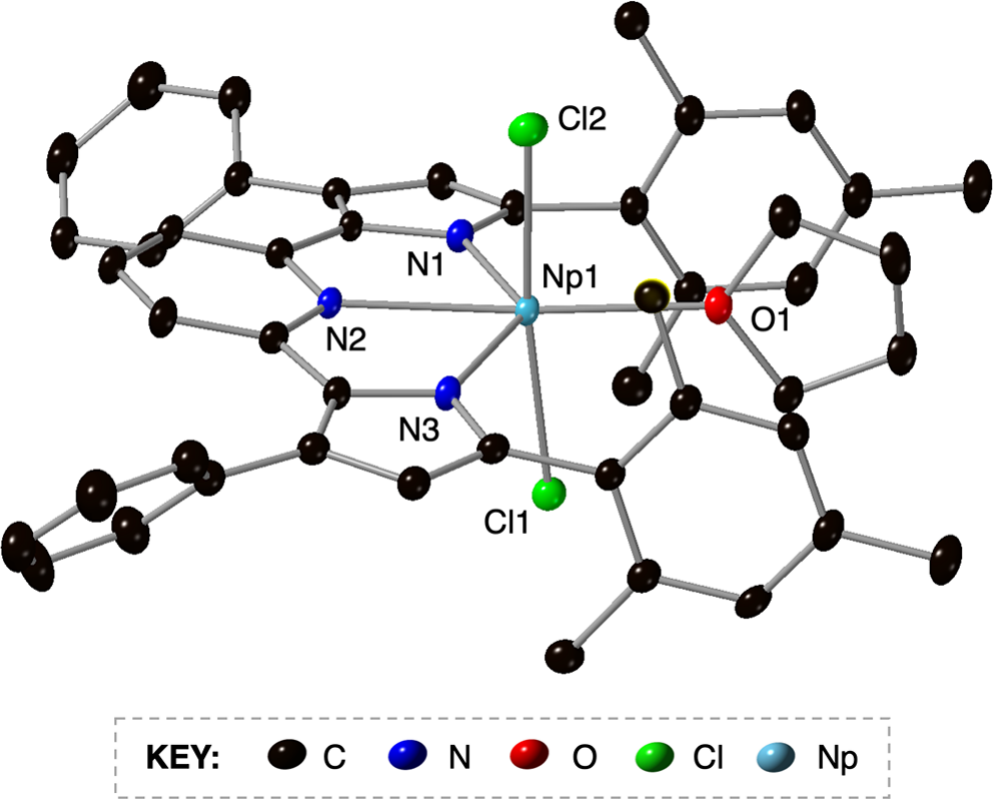
Molecular structure of (^Mes^PDP^Ph^)NpCl_2_(THF) (**1-Np**) is shown with 30% probability ellipsoids. Hydrogen atoms have been omitted for clarity.

To further characterize the product, crystals of **1-Np** suitable for single-crystal X-ray diffraction (SCXRD) were grown from slow diffusion of pentane into a concentrated toluene solution of the product in toluene at −30 °C. Refinement of the data confirmed the structural composition of **1-Np** as the six-coordinate species (^Mes^PDP^Ph^)NpCl_2_(THF) (Figure 2 and Table 1). As observed in the crystal structure of **1-U**, **1-Np** crystallizes in the *Pbcn* space group and displays a distorted octahedral geometry at neptunium. The equatorial plane of **1-Np** is occupied by the (^Mes^PDP^Ph^)^2–^ ligand and a bound THF molecule, with two chloride atoms in axial positions, slightly distorted from linearity (Cl–Np–Cl bond angle = 173.14(4)^o^). The Np–N_pyridine_ distance of 2.465(4) Å is similar to that of **1-U** (U–N_pyridine_ = 2.474(4) Å) but is significantly shortened in comparison to other reported Np–N_pyridine_ bond distances. For example, the Np–N_pyridine_ bond distances reported for NpCl_4_(pyr)_4_ are 2.686(14) Å and 2.688(14) Å and in NpCl_4_(^tBu^Bipy)_2_ range from 2.610(3) to 2.650(3) Å.^34^ Sessler and co-workers have also recently reported an Np–N_pyridine_ bond distance of 2.635(8) Å for a Np(IV) expanded porphyrin complex, Np(IV)[dipyriamethyrin](OTMS)_2_.^13^ Additionally, contraction of the Np–N_pyrrolide_ bond distances in **1-Np** is observed (Np–N_pyrrolide_ = 2.305(4) and 2.304(5) Å) in comparison to the Np porphyrin complex (average Np–N_pyrrolide_ = 2.687(8) Å).^13^ These results indicate that, like the isostructural uranium complex, **1-Np** binds more tightly to the (^Mes^PDP^Ph^)^2–^ ligand compared to other complexes featuring neptunium–nitrogen bonds. The Np–Cl bond distances in **1-Np** (2.568(13) and 2.574(14) Å) are similar to the distances of axially bound chloride atoms in NpCl_4_(THF)_3_ (2.568(2) and 2.575(2) Å)^35^ and shorter than reports of equatorially bound chloride atoms (2.588(9)–2.628(9) Å).^31,34,35^ Overall, the major structural differences between **1-Np**, **1-U**, and the isostructural thorium derivative, (^Mes^PDP^Ph^)ThCl_2_(THF) (**1-Th**), arise due to differences in the ionic radius of the actinide ions (Np < U < Th).

**Table 1:** Selected Bond Distances and Angles for (^Mes^PDP^Ph^)NpCl_2_(THF) (**1-Np**); Distances and Angles for (^Mes^PDP^Ph^)UCl_2_(THF) (**1-U**) and (^Mes^PDP^Ph^)ThCl_2_(THF) (**1-Th**) Are Included for Comparison^28^

complex	(^Mes^PDP^Ph^)NpCl_2_(THF) (1-Np)	(^Mes^PDP^Ph^)UCl_2_(THF) (1-U)	(^Mes^PDP^Ph^)ThCl_2_(THF) (1-Th)
An-Cl	2.5684(13), 2.5741(14) Å	2.5844(13), 2.5927(14) Å	2.6528(10), 2.6566(11) Å
Cl-An-Cl	173.14(4)°	174.04(4)°	172.33(3)°
An-N_pyr_	2.465(4) Å	2.474(4) Å	2.548(3) Å
An-N_pyrrolide_	2.304(5), 2.306(4) Å	2.309(5), 2.301(5) Å	2.362(3), 2.358(4) Å

**Figure 3:**
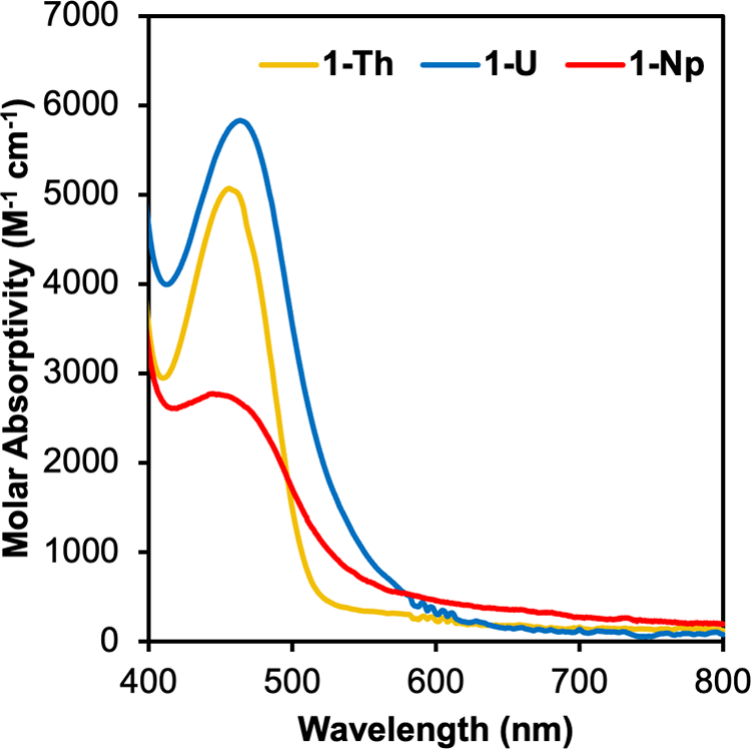
Electronic absorption spectra for (^Mes^PDP^Ph^)NpCl_2_(THF) (**1-Np**), with **1-Th** and **1-U** included for comparison. Spectra were collected at room temperature in dichloromethane.

Previously, we found that the redox-active nature of the (^Mes^PDP^Ph^)^2–^ ligand imparted interesting optical properties on the isostructural (^Mes^PDP^Ph^)MCl_2_(THF) (M = Th(IV), U(IV)) family.^28^ For **1-U**, evidence for LMCT was observed in the electronic absorption spectrum, signaled by low-energy transitions ranging from 400 to 550 nm that extended out to the NIR region, which was corroborated with time-dependent density-functional theory (TD-DFT) calculations in our original report.^28^ In contrast, **1-Th** exhibited exclusively ILCT and LLCT transitions, consistent with the very negative reduction potential and high energy of the 5f orbitals of Th(IV) ions.^30^ These differences in the UV–vis spectra, in combination with the observed color change to dark red upon metalation of the PDP ligand with NpCl_4_(DME)_2_, prompted our interest in the analysis of the optical properties of **1-Np** via electronic absorption spectroscopy (Figure 3). The absorption profile of (^Mes^PDP^Ph^)NpCl_2_(THF) features a relatively intense band at 450 nm (ε = 2750 M^–1^ cm^–1^), which is tentatively assigned to a ligand-to-metal charge transfer (LMCT) transition. The tail of the absorption extends far into the near-infrared region, causing the broadening of the band and resulting in the dark-red color of **1-Np**. Analysis of the near-infrared region of the spectrum of **1-Np** reveals *f-f* transitions, consistent with the assignment of a +4 oxidation state (5*f*^3^ valence electron configuration) of neptunium (Figure S6).^36,37^

The isostructural actinide complexes were synthesized in part to probe the influence of increasing f-electron count on the spectroscopic properties of the target complexes. As referenced previously, **1-Th** contains only LLCT and ILCT transitions, whereas the energetically accessible 5*f* orbitals in **1-U** and **1-Np** (and the reducibility of these metals) result in operative LMCT transitions. The broadening of the absorption bands (and extension into the NIR region) for **1-U** and **1-Np** give rise to their red color, whereas the absence of these features in **1-Th** results in the observed yellow color of the product. There is no discernible trend in the band position in the electronic absorption spectra as the 5*f*-electron count of the An(IV) cation increases (**1-Th** = 458 nm, **1-U** = 470 nm, **1-Np** = 450 nm). The red shift of the absorption maximum for **1-U** compared to **1-Th** is expected due to the lowering in energy of the valence 5*f* orbitals of uranium compared to thorium.^38^ However, the blue shift (∼20 nm) of **1-Np** is surprising, given that it would be expected for the 5*f* orbitals of neptunium to be lower in energy in comparison to that of the uranium(IV) derivative. This suggests that there is likely a larger gap between the 5*f* orbitals of **1-Np** and the LUMO of the PDP ligand, resulting in the observed increase in the energy of the charge transfer band in the electronic absorption spectrum. Computational analysis of the electronic structure of **1-Np** is required to firmly establish the nature of the electronic transitions within the complex but is beyond the scope of this initial report.

**Scheme 2:**
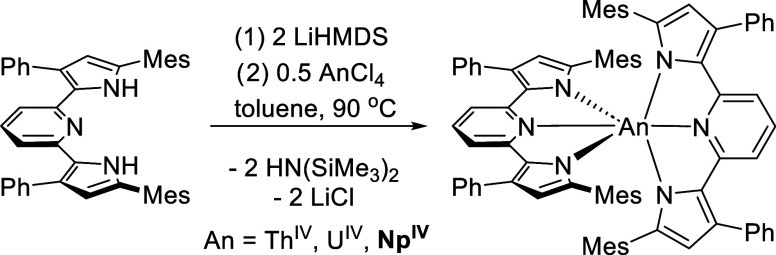
Synthesis of An(^Mes^PDP^Ph^)_2_ Complexes (**1-Th**, **1-U**, **1-Np**)

Next, the formation of a bis-ligated neptunium PDP complex was targeted, as other M(IV) analogues have displayed intriguing electrochemical and spectroscopic profiles.^16,24,25^ Previously, the bis-ligand actinide compounds, M(^Mes^PDP^Ph^)_2_ (M = Th, U), were synthesized.^28^ In this study, it was hypothesized that alkylation of (^Mes^PDP^Ph^)MCl_2_(THF) (M = Th(IV), U(IV)) using benzyl potassium (KCH_2_Ph) would generate basic actinide–carbon bonds that would drive the formation of M(^Mes^PDP^Ph^)_2_ by deprotonation of an additional equivalent of H_2_(^Mes^PDP^Ph^). To avoid having to access the organoneptunium intermediate (which can often have a negative impact on reaction yields), the synthesis of the bis-ligated PDP complex through salt metathesis was pursued, using depleted uranium as a model metal center (see the Experimental Section for details). With a new route to access the bis-ligated actinide complex in hand, an extension of this chemistry to neptunium(IV) was pursued. Addition of one equivalent of Li_2_(^Mes^PDP^Ph^) in toluene to a suspension of half an equivalent of NpCl_4_(DME)_2_ in toluene results in a gradual color change to dark red after heating to ∼90 °C for 6 h. Workup of this product afforded Np(^Mes^PDP^Ph^)_2_ (**2-Np**) in a 70% yield (Scheme 2).

**Figure 4:**
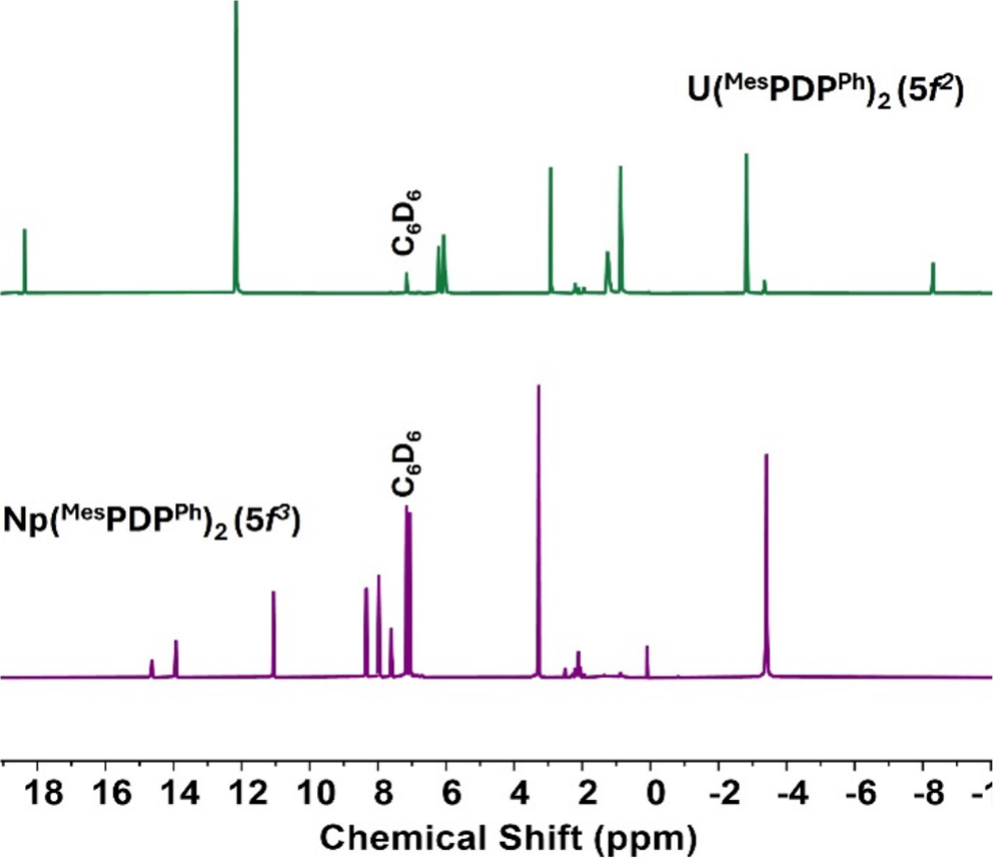
^1^H NMR spectrum (400 MHz) of **2-Np** stacked with **2-U** for comparison was collected in C_6_D_6_ at room temperature (∼21 °C). For full assignments, see the Supporting Information.

The formation of **2-Np** was initially confirmed by ^1^H NMR spectroscopy. Nine paramagnetically shifted resonances ranging from +15 to −4 ppm were observed, consistent with the formation of a *D*_2*d*_-symmetric product in solution (Figure 4). Analysis of the relative integrations in the ^1^H NMR spectrum revealed two prominent resonances at 3.28 and −3.40 ppm with integrations of 12H and 24H, respectively. These signals are assigned to the *para*-methyl and *ortho*-methyl groups of the mesityl substituent of the PDP ligand. The 4-pyridyl proton was located at 14.63 ppm as a well-defined triplet resonance, integrating to two protons. In addition, the signal for the pyrrole protons was located as a singlet at 11.06 ppm. To our surprise, the ^1^H NMR spectrum of **2-Np** (Np(IV), *f*^3^) is significantly different compared to that of **2-U** (U(IV), *f*^2^). In **2-U**, significant deshielding of the *ortho*-methyl protons was observed and attributed to their distance from the paramagnetic 5*f*^2^ ion, where the steric bulk imparted by the PDP ligand forces the mesityl groups closer to uranium. For **2-Np**, the opposite behavior is observed, where the *ortho*-methyl protons are more shielded. Similarly, the *para*-methyl protons for **2-U** are visible at −2.83 ppm in the ^1^H NMR spectrum versus 3.29 ppm for **2-Np**.

To probe this phenomenon, a Np(IV) derivative utilizing a PDP ligand with phenyl substituents in place of mesityl groups, H_2_(^Ph^PDP^Ph^), was targeted in order to investigate the possibility of similar magnetic effects. Salt metathesis between Li_2_(^Ph^PDP^Ph^) and NpCl_4_(DME)_2_ afforded what is hypothesized to be Np(^Ph^PDP^Ph^)_2_ (**3-Np**). The ^1^H NMR spectrum of **3-Np** (Figure S5) displays a similar resonance pattern to **2-Np**, suggesting that the observed paramagnetic shifting is characteristic for these bis(ligand) neptunium complexes and not unique to **2-Np**. Interestingly, it appears that the change in valence f-electrons at the actinide ion within these systems has a significant impact on the local environment of the protons. This suggests different bonding contributions of the 5f orbitals within these systems, though theoretical calculations are necessary to definitively confirm this hypothesis. Though multiple attempts were made to characterize both Np(^Mes^PDP^Ph^)_2_ (**2-Np**) and Np(^Ph^PDP^Ph^)_2_ (**3-Np**) by SCXRD, severe disorder in all suitable crystals precluded the characterization of the bis-ligated product through this method.

**Figure 5:**
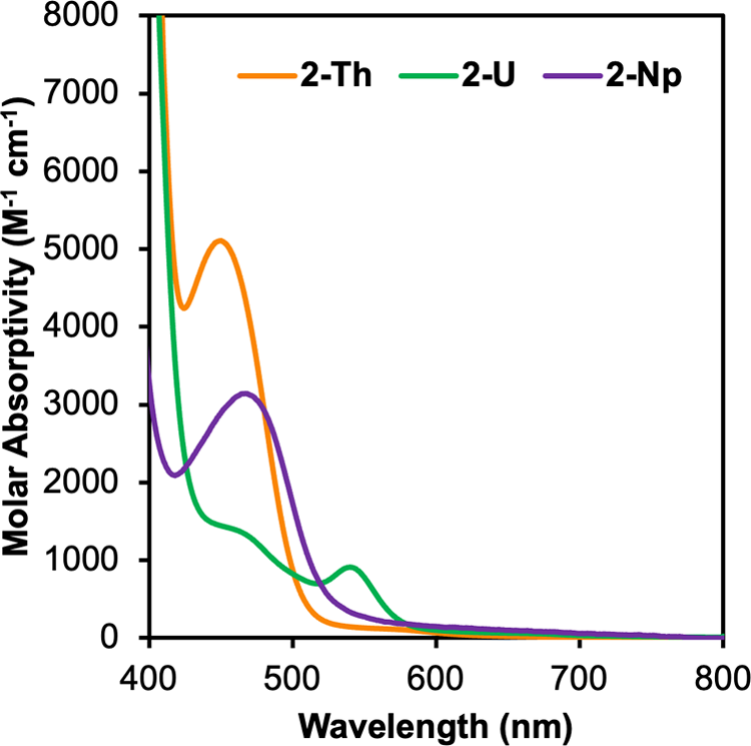
Electronic absorption spectra in the visible region for Np(^Mes^PDP^Ph^)_2_ (**2-Np**), with **2-Th** and **2-U** included for comparison. Spectra were collected at room temperature in dichloromethane.

Analysis of the optical properties of red **2-Np** was performed by electronic absorption spectroscopy (Figure 5). The near-infrared region of the spectrum reveals weak and sharp *f*-*f* transitions, consistent with retention of a + 4 oxidation state of neptunium upon coordination of two PDP ligands (Figure S7). These values are similar to reports of other Np(IV) organometallic complexes.^36,37^ In the visible region, the absorption profile of **2-Np** in DCM displays an intense band at 473 nm (ε = 3250 M^–1^ cm^–1^). There are notable differences upon comparison of the electronic absorption spectra of **2-Th**, **2-U**, and **2-Np** in DCM. Like **1-Th**, **2-Th** displays a single intense band at 452 nm (ε = 5088 M^–1^ cm^–1^) that is assigned as a ligand-based transition, with computational analysis confirming that all filled frontier molecular orbitals are exclusively ligand-centered, with negligible contributions from the metal ion (<3%).^28^ Interestingly, the position of the absorption band for **2-Np** is red-shifted ∼20 nm compared to **2-Th**, and it displays a similar intensity. The band positions of **2-Th** and **2-Np** closely resemble each other, with the most notable difference being the increase in bandwidth in **2-Np** that results in an extension of absorption to ∼600 nm, responsible for the red color. The observed similarities between the thorium and neptunium complexes suggest that the electronic transition for **2-Np** may contain some ligand-based charge transfer character. However, the extension of the band to lower energies, ascribed to ligand-to-metal charge transfer character in our original report, indicates that the band in **2-Np** may also contain some LMCT character and that the electronic transitions within **2-Np** are complicated.

There are significant differences when analyzing the electronic absorption spectrum of **2-U** compared to **2-Np**. Two bands are observed at 466 nm (ε = 1322 M^–1^ cm^–1^) and 545 nm (ε = 887 M^–1^ cm^–1^), consistent with low-energy LMCT transitions calculated with TD-DFT in our original report for the U(IV) complex.^28^ It is noted that low-energy transitions in the visible region are not observed experimentally for **2-Np** apart from the band at 473 nm, although it was originally hypothesized that these transitions should be more readily facile to access, given the likelihood of the increased orbital overlap between the π orbitals of the PDP ligand and the lower energy 5f orbitals of the Np ion. This would theoretically result in a lower energy LMCT state and a red shift in the absorption spectrum for **2-Np**. As such, it is possible that the band at 473 nm corresponds to a ligand-to-metal charge transfer transition that is more favorable (more “allowed”) compared to the low-intensity LMCT transitions in **2-U**. The results herein demonstrate that the electronic transitions of **2-Np** are convoluted due to the 5*f*^3^ electronic configuration of the metal ion and likely require computational analysis to fully unravel the complicated electronic transitions.

The photophysical properties of bis-ligand PDP complexes (e.g., Sn^IV^(^Me^PDP^Ph^)_2_ (5s^0^5p^0^),^16^ Zr^IV^(^Mes^PDP^Ph^)_2_ (4*d*^0^),^25^ and Hf^IV^(^Mes^PDP^Ph^)_2_ (5*d*^0^)^29^) have garnered attention due to their long-lived triplet excited states and high quantum efficiencies. More recently, this series was extended to **2-Th** (5*f*^0^) and **2-U** (5*f*^2^) to investigate the role of *f*-electrons in emission processes.^28^**2-Th** possessed strong room-temperature photoluminescence with a high quantum yield (Φ_PL_ = 42%) and long-lived excited state (τ = 0.304 ms), whereas **2-U** was not luminescent. The emission decay pathway in **2-U** was hypothesized to proceed through the 5f orbital manifold upon excitation into the LMCT band, resulting in a quenching of emission. Due to the lowering in energy of the 5f orbitals for neptunium compared to thorium and uranium, it was hypothesized that **2-Np** (5*f*^3^) would also be nonluminescent like **2-U**. Analysis of a sample of **2-Np** under ultraviolet irradiation showed no emission from the complex, which affirms this hypothesis.

**Figure 6:**
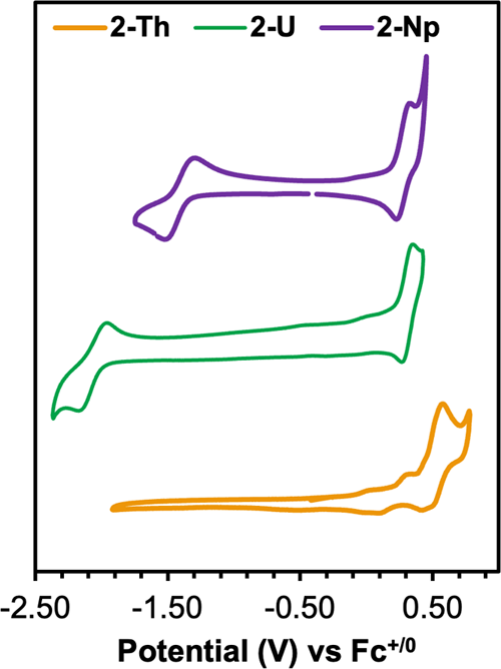
Cyclic voltammograms of Th(^Mes^PDP^Ph^)_2_, U(^Mes^PDP^Ph^)_2_, and Np(^Mes^PDP^Ph^)_2_ recorded in DCM (1 mM analyte, 0.1 M TBAPF_6_, scan rate = 200 mV s^–1^).

Having established a thorough understanding of the solution-phase and solid-state structures of both **2-U** and **2-Np**, the electrochemical properties of these compounds using cyclic voltammetry (CV) were investigated (Figure 6). It is noted that due to the electrochemical instability of **1-U** under oxidizing conditions (Figure S8), the poorly resolved cyclic voltammogram precluded electrochemical analysis of **1-Np**. Thus, the electrochemistry of **1-U** and **1-Np** is excluded from this study. However, it was hypothesized that the steric bulk imparted by the coordination of two PDP ligands to the actinide would increase the stability of the complexes, allowing for analysis of their electrochemical properties. Accordingly, CV measurements of U(^Mes^PDP^Ph^)_2_ and Np(^Mes^PDP^Ph^)_2_ were conducted on 1 mM solutions of the compounds in DCM with 0.1 M TBA(PF_6_) and referenced to Fc^+/0^. For both **2-U** and **2-Np**, scanning anodically revealed a reversible feature at 0.317 V (**2-U**) and 0.298 V (**2-Np**). Due to the similarity in potential, these redox events are assigned as oxidation of the PDP ligand. This is further supported by the presence of a similar redox event in the CV of ^Mes^PDP^Ph^UO_2_(THF) (0.30 V vs Fc^+/0^),^27^ where uranium is already in its highest possible oxidation state, supporting ligand-based oxidation. The oxidative event in **2-Np** is shifted 20 mV relative to that of **2-U**, suggesting that upon coordination of Np(IV), the PDP chelate is more readily oxidized. The cyclic voltammogram of **2-Th**, in comparison, contains a pseudoreversible oxidative event at 0.521 V vs Fc^+/0^ (Figure 5). This oxidation is expected to be exclusively ligand-based due to the similarity in potential to **2-U** and **2-Np**, coupled with the lack of accessible 5f-electrons in Th(IV) (5*f*^0^).

When scanning cathodically, a reversible reduction event is observed in **2-U** centered at −2.06 V (vs Fc^+/0^) and at −1.41 V (vs Fc^+/0^) for **2-Np**. These events are assigned as U(IV) → U(III) and Np(IV) → Np(III) reductions due to these potentials being in the expected range for these processes.^9^ Notably, the reduction is shifted anodically by 0.65 V for **2-Np** relative to **2-U**, consistent with other reports of An(IV)/An(III) redox couples for isostructural uranium and neptunium complexes. For example, the redox potentials of An(IV)/An(III) couples in a series of Cp^R-^ complexes, Cp_4_An, Cp_3_AnCl, and Cp*_2_AnCl_2_, were measured and showed more modest reduction potentials in the neptunium derivatives compared to the uranium complexes.^39^ This indicates that **2-Np** can be readily reduced and the Np(III) compound should be accessible chemically with mild reducing agents.

## Conclusions

In summary, we have reported the synthesis of two new neptunium(IV) pyridine dipyrrolide complexes, ^Mes^PDP^Ph^NpCl_2_(THF) (**1-Np**) and Np(^Mes^PDP^Ph^)_2_ (**2-Np**). These derivatives were made in moderate yields and fully characterized using a variety of spectroscopic techniques and, where possible, X-ray crystallography. The steric bulk imparted by the PDP chelate results in a distorted octahedral geometry around the neptunium center in the solid-state structure of **1-Np**. Neptunium oxidation states were confirmed using electronic absorption spectroscopy, with data showing both species in the +4 oxidation state. The electronic absorption spectra of these compounds feature intense charge transfer bands that are hypothesized to be a mixture of ligand-to-metal charge transfer and ligand-based transitions. In DCM solution, U(^Mes^PDP^Ph^)_2_ and Np(^Mes^PDP^Ph^)_2_ display rich electrochemistry with reversible ligand-based oxidative chemistry and An(IV)/(III) reduction couples, suggesting that reduced analogues of the bis-ligated complexes may be chemically isolable. Notably, systematic shifting toward anodic potentials is observed for the neptunium complex compared to the uranium complex, indicative of the overall ease of reducibility of the Np(IV) ion compared to U(IV). With the thorium and uranium analogues being previously synthesized, we have begun to develop a series of PDP-actinide compounds that allow us to probe the influence of increasing f-electron count on their spectroscopic and electrochemical properties: Th(IV), *f*^0^, U(IV), *f*^2^, and Np(IV), *f*^3^. Future studies will focus on the reactivity of these species as well as isolation of low-valent derivatives.

## Supporting Information Available

Additional spectroscopic, crystallographic, and voltammetric data (PDF)
