# Supplementary material for: Neptunium Pyridine Dipyrrolide Complexes
Source: Organometallics. 2025 Jan 9;44(2):439–46. doi: 10.1021/acs.organomet.4c00472 (PMC11776103; doi:10.1021/acs.organomet.4c00472)
Supplement: Supplementary file 1 — om4c00472_si_001.pdf [file om4c00472_si_001.pdf]

Electronic Supporting Information

Neptunium Pyridine Dipyrrolide Complexes

Leyla R. Valerio[1], Andrew W. Mitchell[2], Lauren M. Lopez[2], Matthias Zeller[2], Suzanne C. Bart*[2], Ellen M. Matson*[1]

[1] Department of Chemistry, University of Rochester, Rochester NY 14627 USA

[2] H. C. Brown Laboratory, James Tarpo Jr. and Margaret Tarpo Department of Chemistry, Purdue University, West Lafayette, IN 47907, USA

Corresponding Author Contact Information:

Suzanne C. Bart: sbart@purdue.edu

Ellen M. Matson: matson@chem.rochester.edu

Table of Contents

1. ^{1}H NMR Spectra

Figure S1. ^{1}H NMR spectrum of **1-Np** in C_6D_6……………………………………………..…………..3
Figure S2. ^{1}H NMR spectrum of **1-U** in C_6D_6…………………………………………………………….3
Figure S3. ^{1}H NMR spectrum of **2-Np** in C_6D_6…………………………………..………………..…4
Figure S4. ^{1}H NMR spectrum of **2-U** in C_6D_6……………………………………….……………….…4
Figure S5. ^{1}H NMR spectrum of **3-Np** in C_6D_6………………………...………………………….…5

2. Single Crystal X-ray Diffraction

Table S1. Crystallographic parameters for ($^{Mes}PDP^{Ph}$)$NpCl_2$(THF) **1-Np** …………………………..…6

3. Electronic Absorption Spectroscopy

Figure S6. Electronic absorption spectrum (NIR region) of **1-Np**…………………………………….....7
Figure S7. Electronic absorption spectrum (NIR region) of **2-Np**…………………………………….....7

4. Electrochemistry

Figure S8. Cyclic voltammogram of **1-U**…………………………………………………………….…8

1. ^{1}H NMR spectra

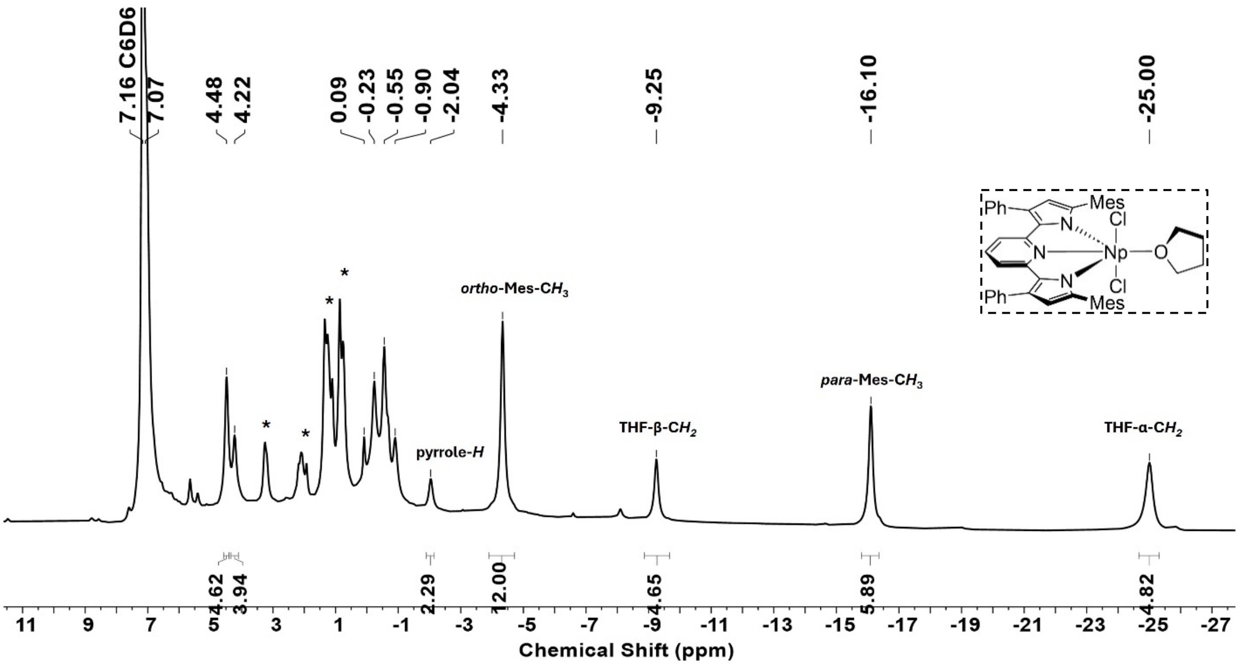

Figure S1. ^{1}H NMR spectrum of **1-Np** in C_6D_6 collected at 21 °C. Peaks with asterisks (*) correspond to residual solvent present in the sample.

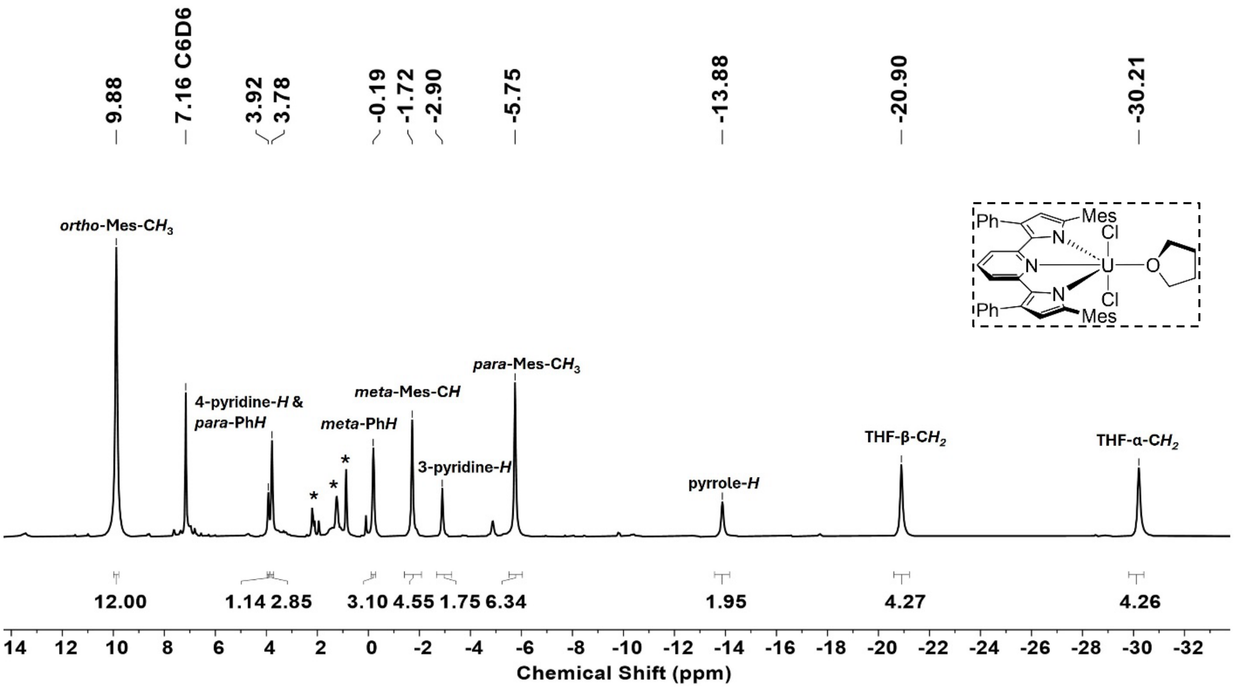

Figure S2. ^{1}H NMR spectrum of **1-U** in C_6D_6 collected at 21 °C. Peaks with asterisks (*) correspond to residual solvent present in the sample.

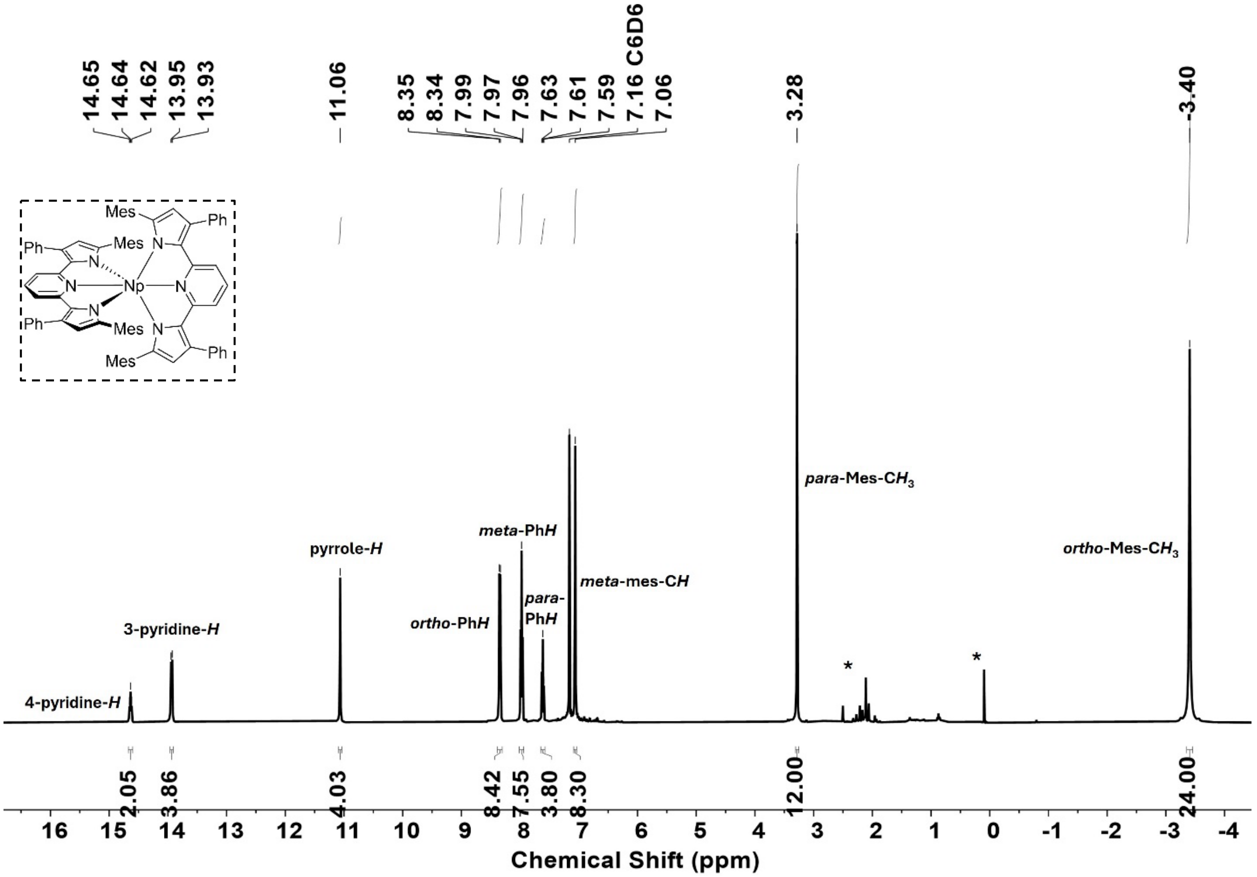

Figure S3. ^{1}H NMR spectrum of **2-Np** in C_6D_6 collected at 21 °C. Peaks with asterisks (*) correspond to residual solvent and grease present in the sample.

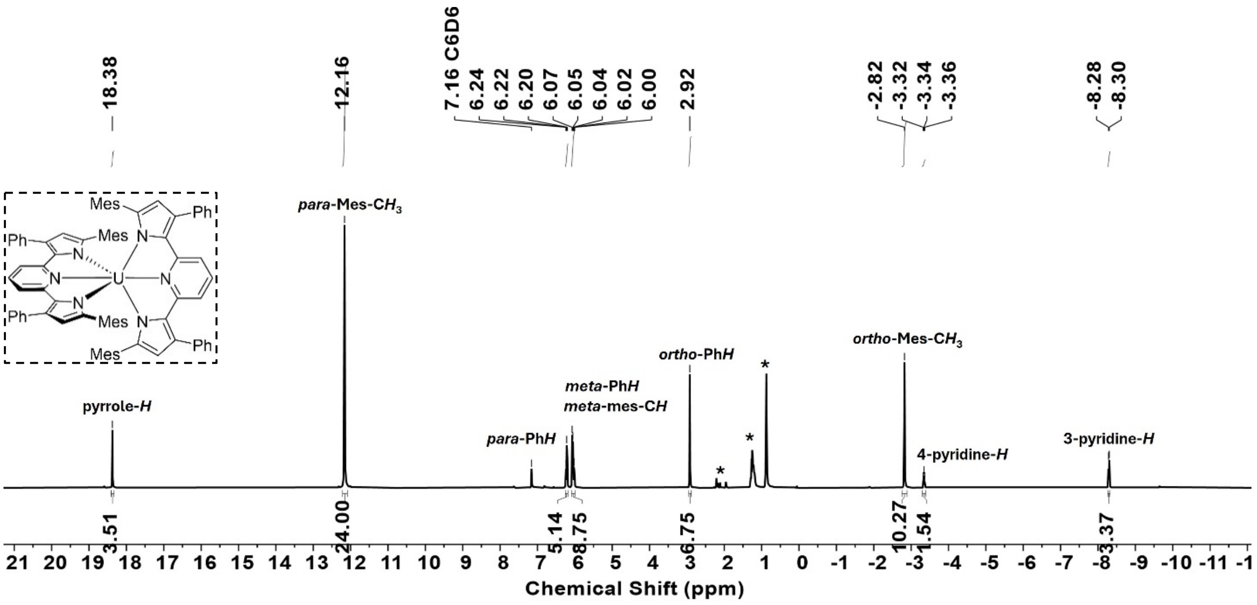

Figure S4. ^{1}H NMR spectrum of **2-U** in C_6D_6 collected at 21 °C. Peaks with asterisks (*) correspond to residual solvent present in the sample.

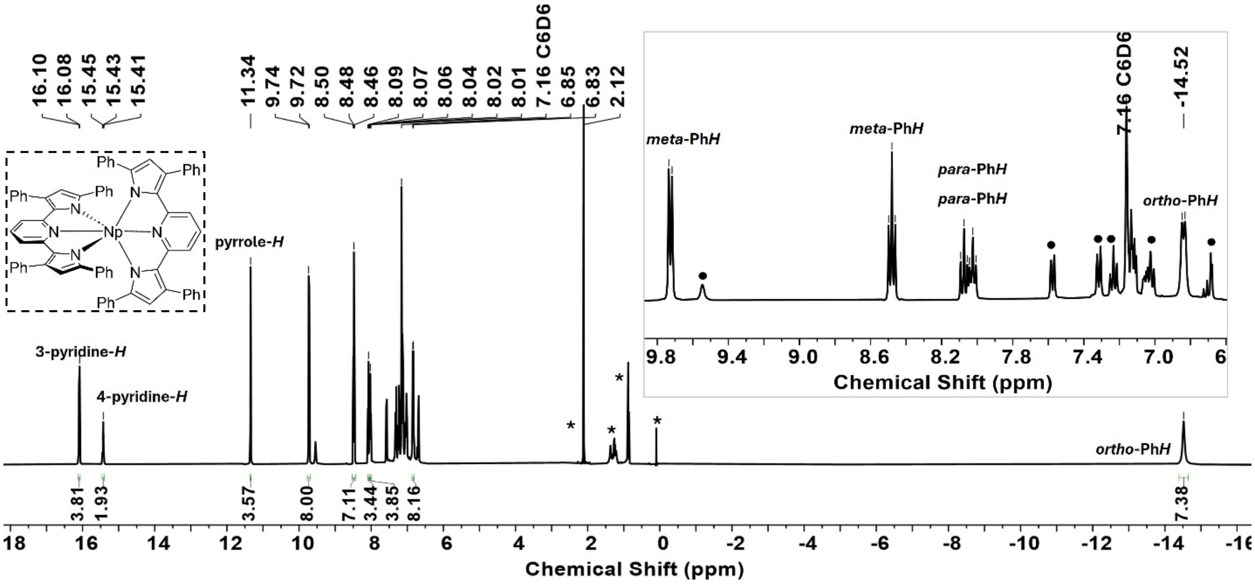

Figure S5. ^{1}H NMR spectrum of **3-Np** in C_6D_6 collected at 21 ^oC. Peaks with asterisks (*) correspond to residual solvent present in the sample. Unlabeled peaks with circles correspond to a small amount of $H_2(^{Ph}PDP^{Ph})_2$ present in the sample.

2. Single Crystal X-ray Diffraction

Table S1. Crystallographic parameters for (^{Mes}PDP^{Ph})NpCl_2(THF) **1-Np**

**Empirical Formula**	$C_{115}H_{114}N_6Cl_4Np_2O_2$
**Formula Weight**	2227.92
**Temperature**	150(2)
**Wavelength**	CuKα (λ = 1.54178)
**Crystal System**	orthorhombic
**Space Group**	Pbcn
**Unit cell Dimensions**	a = 31.0875(16)<br>b = 19.8688(10)<br>c = 15.8212(8)<br>α = 90°<br>β = 90°<br>γ = 90°
**Volume/Å^3**	9772.3(9)
**Z**	4
**Reflections Collected**	147081
**Independent Reflections**	10681
**Goodness-of-Fit on F^2**	1.067
**Final R indices [I>2sigma(I)]**	R_1 = 0.0390, wR_2 = 0.1046

3. Electronic Absorption Spectroscopy

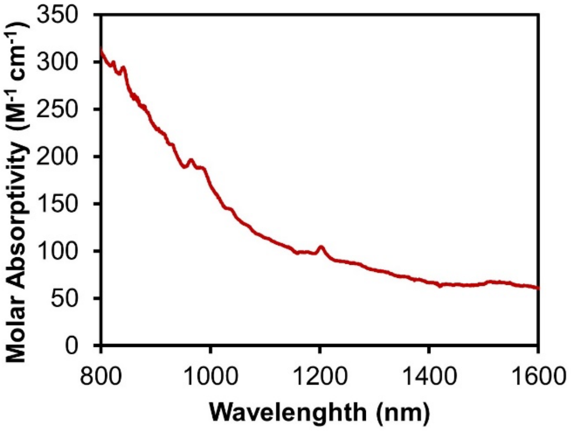

Figure S6. NIR region of the electronic absorption spectrum for (^{Mes}PDP^{Ph})$NpCl_2$(THF) (**1-Np**), collected at room temperature in dichloromethane.

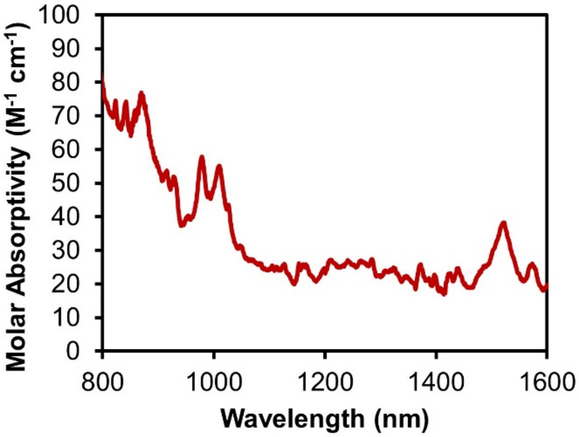

Figure S7. NIR region of the electronic absorption spectrum for Np(^{Mes}PDP^{Ph})$_2$ (**2-Np**), collected at room temperature in dichloromethane.

4. Electrochemistry

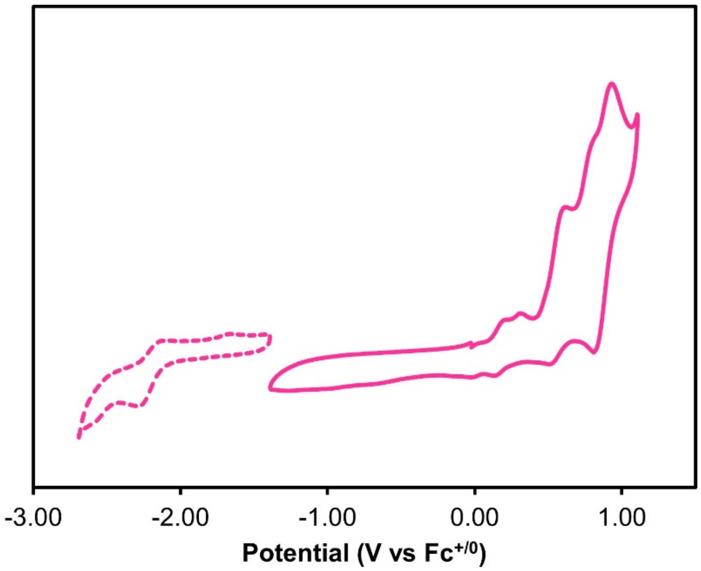

Figure S8. Cyclic voltammogram of **1-U** in DCM (0.1 M TBA(PF_6) at 200 mV s^{-1}). The solid line is oxidative scans and the dashed line is reductive scans. Further cycling results in degradation of the sample.
